# Lipid liquid-crystalline nanoparticles as a suitable platform for accommodating sensitive membrane proteins: monitoring the activity of HMG-CoA reductase

**DOI:** 10.1186/s12951-025-03370-6

**Published:** 2025-05-07

**Authors:** Michalina Zaborowska-Mazurkiewicz, Ewa Nazaruk, Renata Bilewicz

**Affiliations:** University of Warsaw, Faculty of Chemistry, Pasteura 1, 02093 Warsaw, Poland

**Keywords:** Lipid nanoparticles, *HMG-CoA* reductase, *NADPH*, Transmembrane enzyme, Cyclic voltammetry, Bioelectrochemistry, Hexosome

## Abstract

Biological molecules such as integral membrane proteins, peptides, and nucleic acids that are not soluble or sufficiently stable in aqueous solutions can be stabilized through encapsulation in lipid nanoparticles. Discovering the potential of lipid liquid-crystalline nanoparticles opens up exciting possibilities for housing sensitive membrane proteins. Lipid mesophases provide an environment that protects the cargo, usually a drug, from rapid clearance or degradation. This study employed the mentioned platform to stabilize a different cargo—an essential transmembrane enzyme, *HMG-CoA* reductase (*HMGR*). The nanostructured lipid liquid-crystalline (*LLC*) nanoparticles known as hexosomes are selected as a convenient nanocontainer for the redox–active protein for real-time monitoring of its functions in the bulk of the solution and point to the applicability of the proposed platform in the evaluation of therapeutic functions of the protein by standard physicochemical methods. Instead of using detergents, which usually affect the functions and stability of sensitive membrane proteins, we provide a suitable environment, protecting them in the bulk of the solution against other present species, e.g., toxic compounds or degrading proteins. The objective was to optimize the composition and structure of the lipid nanoparticles to meet the needs of such sensitive and flexible membrane proteins as *HMGR* and compare the functioning of the encapsulated enzyme with that of the same protein free in the aqueous solution. The catalytic reaction of *HMGR* involves the 4-electron reduction of *HMG-CoA* to mevalonate and *CoA* while simultaneously oxidizing *NADPH* to *NADP*^+^. Subsequently, mevalonate is transformed into cholesterol. The hexosomes we selected as lipid nano-containers were composed of monoolein, 1-oleoyl-rac-glycerol (*GMO*), Pluronic^®^ F127, and poly(ethylene glycol) (*PEG*). These specific structural characteristics of the lipid nanoparticles were found optimal for enhancing the stability of *HMGR*. We characterized these hexosomes using dynamic light scattering (*DLS*), small-angle X-ray scattering (*SAXS*), and cryogenic electron microscopy (Cryo-TEM) methods, both with and without the encapsulated protein. In our innovative approach, the enzyme activity was assessed by monitoring changes in *NADPH* concentration outside the nanocarrier. We tracked fluctuations in *NADPH* levels during the catalytic reaction using two independent methods: *UV–Vis* spectrophotometry and cyclic voltammetry. Significantly, we could demonstrate the inhibition of the nano-encapsulated enzyme by fluvastatin, an enzyme inhibitor and cholesterol-lowering drug. This paves the way for the discovery of new enzymatic inhibitors and activators as therapeutic agents controlling the activity of membrane proteins, thereby inspiring future cholesterol-lowering therapies in our case and, in general, further research and potential new treatments.

## Introduction

The encapsulation of membrane proteins, peptides, and nucleic acids in lipidic nanoparticles has been demonstrated to stabilize the unstable, delicate, or poorly soluble biologically significant molecules and/or facilitate their effective delivery to the organism [1–5]. Protein therapeutics are complicated due to size, rate of degradation of membrane proteins in vivo, low permeation through biological barriers, pH, and temperature sensitivity. Proteins require frequent administration to preserve the necessary therapeutic levels in vivo due to their short half-lives. Formulation strategies combining proteins with lipid liquid-crystalline carriers such as cubosomes and hexosomes show potential for improving bioavailability while preserving protein activity and facilitating their transfer across the body's barriers. Encapsulating protein in long-lasting injectable delivery systems can improve protein therapeutics by prolonging their functions and reducing the need for repeat interventions [3, 6, 7].

In the case of membrane proteins, the lipidic mesophases constitute a matrix resembling their natural environment in the cell, which facilitates the studies of, e.g., protein or peptide interactions with drugs that most often target the catalytic site of a given enzyme [8]. Many of the lyotropic properties of liquid crystalline phases occur in nature, making them structures of interest and significance in biomimetic nanomaterials engineering, pharmacy, medicine, and biology. The lipidic nanoparticles studied in this paper resemble the topology of some membrane nanostructures found in eukaryotic cell organelles—mitochondria and the endoplasmic reticulum [9].

Monoolein is a suitable lipid for forming liquid crystalline phases due to its moderately low hydrophilic—lipophilic balance (*HLB*) and critical packing parameter (*CPP*) above 1 [10]. Lyotropic liquid crystals (*LCs*) can assume various configurations, lamellar phase (*L*_*α*_), micellar (*L*), inverse hexagonal phase (*H*_*II*_), or bicontinuous cubic phase (*Q*_*II*_), [11–13] which can be assessed based on structural studies using small-angle X-ray scattering (*SAXS*) [14]. In the inverse hexagonal phase (*H*_*II*_), amphiphiles form micellar cylinders arranged approximately in a hexagonal lattice. The liquid crystalline lipid mesophases and their dispersed forms, cubosomes or hexosomes, are characterized by a homogeneous system of water channels with the possibility of adjusting the width of the pores to the sizes of the incorporated biomolecules.

The embedding of transmembrane proteins within the cubic phase has been proven an effective method for crystallizing essential membrane proteins for high-resolution structure determination [15, 16]. However, limited studies specifically address these proteins’ functions and activities when immobilized in this biocompatible environment. Notably, research has been conducted on the activities of various proteins, including photosynthetic protein transporters [17], heme-copper oxidases [18], caa3-type cytochrome oxidase [19], and the enzyme DgaK within the lipid cubic phases [19, 20]. The lipid mesophase is a porous, liquid crystalline medium that resembles a molecular sponge with a lipid bilayer structure and aqueous channels. Water-soluble substances can quickly diffuse in and out of these channels, which connect to the surrounding bulk medium. We have used the monoolein-based liquid crystalline cubic phase as a matrix for soluble oxidases [21] and membrane-bound enzymes or membrane proteins [22, 23]. The ion channel, *OmpF* porin, was reconstituted in the bilayers of a Pn3m bicontinuous cubic and hexagonal phases, creating interconnections between two sets of aqueous channels and enabling pH-controlled molecular gating [24, 25]. The *EcCLC* antiporter was studied in the bicontinuous cubic phase [26]. We investigated chloride ion transport in reconstituted thin cubic phase films of EcCLC using electrochemical methods and all-atom molecular dynamics (*MD*) simulation, examining the relationship between ion flux, applied electrode potential, and pH [27].

Lipid-based nanoparticles are of considerable interest for the functional investigation of membrane proteins, which play crucial roles in various cellular processes. Liposomes have been used to study potassium channel [28] and ion channel *KvLm* activities [29]. Cubosomes prepared via a liquid precursor method were used to measure the activity of the soluble proteins [3]. Cubosomes are more stable than liposomes and are better at encapsulating hydrophobic compounds [30]. They can transport a variety of proteins, nucleic acids, and peptides [31–36]. These unique capabilities enable the stable confinement of multiple proteins, as well as addressing them to their intended biological targets without degradation by enzymes. Nanodispersions of cubosomes, micellar cubosomes, and hexosomes stabilized by nonionic block co-polymer Pluronic^®^ F127 and poly(ethylene glycol) (*PEG*) derivatives are of special interest in drug delivery, allowing for improvement in their bioavailability, optimization of their circulation in the body, and the control of fate-time after in vivo administration [37–41]. In the present paper, we have demonstrated the efficacy of the hexagonal phase (*PEG*)ylated nanoparticles as valuable tools for encapsulating and investigating the activity of membrane proteins. Our study employed electrochemical methods alongside UV–Vis spectrophotometry to evaluate the activity and inhibition of the membrane protein.

**Scheme 1:**
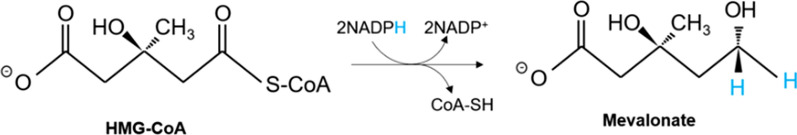
Rate-limiting reaction in cholesterol biosynthesis catalyzed by *HMG-CoA* reductase

The membrane protein used in this study is a key enzyme in cholesterol biosynthesis —3-hydroxy-3-methylglutaryl coenzyme A reductase (*HMGR*). It catalyzes the limiting step reaction in this process, the reduction of *HMG-CoA* to mevalonic acid with the simultaneous oxidation of *NADPH* to *NADP*^+^ (Scheme 1) [42–44]. The proper functioning of this enzyme directly determines cholesterol levels in the blood. The excess of LDL cholesterol (known as “bad cholesterol”) causes the accumulation of fat plaques in blood vessels, which increases the risk of heart disease and stroke [45]. *HMGR* is a transmembrane enzyme in the membranes of the endoplasmic reticulum in the human body, so its natural environment is the lipid environment [42, 46]. It has a hydrophobic part that repeatedly intertwines the lipid membrane and a hydrophilic catalytic part with an affinity for the cell cytosol. The dimensions of the portion of the enzyme bound to the lipid bilayer (in the case of human *HMGR*) are approximately 8 × 5 × 5 nm. In comparison, the catalytic region (substrate binding pocket) measures about 4 × 4 nm [42, 47].

One way to regulate excessive cholesterol production is to use *HMG-CoA* reductase inhibitors, statins [48]. Due to the remarkable similarity in the structure of statins to *HMG-CoA*, they can compete for the catalytic site of the reductase [49] Statins reduce the rate of catalysis by lowering the number of enzyme molecules binding to the substrate. Still, it should be noted that significantly higher substrate concentration can eliminate the effect of inhibitors. Recently, we studied the activity of *HMGR* in Langmuir lipid layers at the air/water interface using UV–Vis spectrophotometric and electrochemical measurements [50].

In the present paper, we demonstrate the efficacy of the hexagonal phase as a valuable tool for investigating the activity of the membrane protein. We describe the structural changes in *GMO/Pluronic®F127/PEG* hexosomes following enzyme incorporation using dynamic light scattering (*DLS*), small-angle X-ray scattering (*SAXS*), and cryo-transmission electron microscopy (*Cryo-TEM*). Using electrochemical and UV–Vis methods, we answer the following question: can a lipidic hexosome serve as a nanocontainer for *HMGR*, offering satisfactory stability while maintaining similar activation/inhibition reactions as in the original biological environment.

## Experimental

### Materials

Monoolein 1-oleoyl-rac-glycerol (*GMO*), Pluronic®F127 (also known as Poloxamer 407), and poly(ethylene glycol) (*PEG*) used in the preparation of the hexosomes, and the chemicals needed to measure the activity of transmembrane enzyme (*HMG-CoA* reductase (*HMGR*, 76 kDa with purity ≥ 90% according to SDS-PAGE), 3-hydroxy-3-methylglutaryl coenzyme A (*HMG-CoA*), nicotinamide adenine dinucleotide phosphate (*NADPH*), 2,2-azino-bis-(3-ethyl-benzthiazoline-6-sulfonic acid) (*ABTS*) and fluvastatin were all purchased from Merck, Poland. The *HMGR* enzyme solution sample contained the protein in 50 mmol/L Tris buffer, pH 7.5, 5 mmol/L dithiothreitol solution, 1:200 Protease Inhibitor Cocktail, and 50% (w/v) glycerol. The catalytic reaction was performed in 10 mmol/L PBS buffer, pH 7.4 prepared using ultrapure water (MilliQ) (pH = 6.998, 18.2 MΩ·cm; Millipore, USA). The BCA Assay kit (Merck, Poland) was used to determine the bound/unbound enzyme amount. The *HMG-CoA* Reductase Assay Kit (Merck, Poland) was, in turn, used to determine *HMGR *activity based on *NADPH* levels.

### Hexosome preparation

**Scheme 2:**
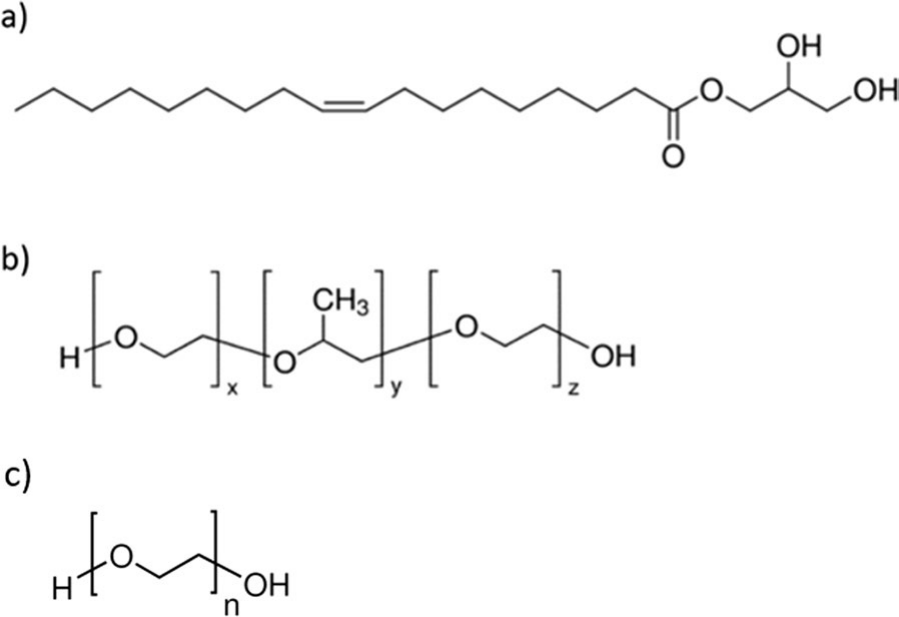
Chemical structures of a) monoolein 1-oleoyl-rac-glycerol (*GMO*) and b) polymer Pluronic*®*F127 (x = 95, y = 62, z = 95), and c) poly(ethylene glycol) (*PEG*) (n = 200)

Lipid nanoparticles incorporating human *HMGR* were prepared following Rizwan et al.’s procedure using a liquid precursor mixture [35]. This liquid precursor (*LP*) mixture consisted of lipid monoolein (*GMO*), a stabilizing agent (Pluronic®F127), and a hydrotrope, polyethylene glycol (*PEG*) (Scheme 2). To create the *LP* mixture, 100 mg of monoolein, 15% w/w of Pluronic®F127 (with respect to the monoolein), and 70% w/w of *PEG* were dissolved in excess chloroform to form a homogeneous solution. The chloroform was then evaporated under a stream of argon and left overnight under reduced pressure. Subsequently, 50 μL of *HMGR* solution (1 mg/mL) was added to the *LP* mixture and vortexed to ensure thorough mixing (the concentration of *HMGR* was measured using the BCA assay, yielding a result of 1 mg/mL). A phosphate buffer was added to the *LP*, and the samples were vortex-mixed. 1 mL of phosphate buffer was added to the *LP*, and the samples were vortex-mixed. An aliquot (500 µL) of lipid dispersion was centrifuged for 30 min at 14,000 rpm (RCF = 14682 × g) to separate the unbound enzyme from the entrapped protein fraction. The supernatant was then analyzed using the BCA assay. To minimize the possible interference from lipids, 2% sodium dodecyl sulfate was added to the samples before analysis. The entrapment of *HMGR* was calculated by determining the difference between the total amount of the protein added and the free fraction present in the supernatant.

### Methods

#### Small-angle X-ray scattering (SAXS)

Small-angle X-ray scattering (*SAXS*) measurements were conducted utilizing a Bruker Nanostar system, which incorporates a Cu Kα X-ray source (*λ* = 1.54 Å) and a Vantec 2000 area detector. The samples were housed in 1.5 mm capillaries from Hampton Research and maintained at a consistent temperature of 24°C throughout the measurement process. Before the *SAXS* analysis, the samples were allowed to equilibrate overnight at room temperature. The obtained two-dimensional diffraction pattern was transformed into a one-dimensional scattering function, denoted as *I(q)*, where *q* (nm^−1^) represents the length of the scattering vector. We subsequently compared the *q* values of the observed peaks to known Miller indices corresponding to various mesophases to determine the phase identity. Lattice parameters for each phase were calculated based on the position of the peaks in the 1D scattering curves using the following equations:

1 $${\text{a}}_{\text{hex}}= \frac{4\uppi }{\sqrt{3} {\text{q}}_{0}} \times \sqrt{{\text{h}}^{2}+{\text{k}}^{2}+\text{hk}}$$

where *a*_*hex*_ is the lattice parameter of hexagonal phase, h and k are Miller indices of the Bragg peak, and “*q*” is the scattering vector.

The radius of the water channel was calculated using the formula:

2 $${\text{r}}_{{{\text{w}}_{{{\text{hex}}}} }} = {\text{a}}\sqrt {\varphi_{{\text{w}}} \frac{\sqrt 3 }{{2\pi }}}$$

where *r*_*w hex*_ is the radius of the water channel for the hexagonal phase, *a* is the lattice parameter and $\varphi$_*w*_ is the water weight fraction.

#### Cryo-transmission electron microscopy (Cryo-TEM)

Here, 3 µL of hexosome dispersions (without and with *HMGR*) were plunge-frozen onto glow-discharged Quantifoil Au R1.2/1.3 holey carbon grids with 2 nm ultrathin carbon using a Thermo Fisher Vitrobot Mark IV with the following settings: blot time 4 s, blot force 4, waiting time 10 s. Two-dimensional electron cryomicroscopy images were taken on a Thermo Fisher Glacios TEM operating at 200 kV, equipped with a 4 k × 4 k Falcon 3EC direct electron detection camera at a magnification of 92 k, which corresponds to a pixel size of 1.587 Å at the specimen level. The total electron dose was approximately 50 e/Å^2^.

#### Dynamic light scattering (DLS)

The hydrodynamic diameter measurement for hexosomes (diluted 50-fold in PBS buffer) was performed using the DLS method (scattering angle 173°) with a Zetasizer Nano ZSP (Malvern Panalytical, Malvern, UK). Additionally, zeta potential measurements (in water) were performed to determine changes in the surface potential of the obtained structures based on the Helmholtz—Smoluchowski equation.  [51, 52] The DLS measurement was performed in quartz cuvettes, while the zeta potential was assessed in specially adapted cuvettes with electrodes (Malvern Panalytical, Malvern, UK); both measurements were performed at 25 ± 1 °C.

#### UV–Vis Spectrophotometry

Spectrophotometric measurements showing the enzyme activity were performed by recording spectra in the wavelength range from 220 to 500 nm and recording the maximum absorbance value at 340 nm (characteristic for *NADPH*). Measurements were performed on a Synergy LUX Multimode spectrophotometer (BioTek Inc., USA) in the microplates. The reference measurement in this reaction was done according to the manufacturer's (Merck, Poland) protocol, where the final concentrations of the individual components were 0.4 mmol/L *NADPH*, 0.2 mmol/L *HMG-CoA*, and 4·10^–8^ mol/L *HMGR*. The enzyme activity was determined using the equation provided by the manufacturer (Merck, Poland) [53]:

3 $$Units/mgP=\frac{{(\Delta A}_{340/sample}-{\Delta A}_{340/control})\cdot 0.3 }{12.44 \cdot V \cdot 0.6 \cdot 0.55}$$

where *ΔA*_*340*_ is the absolute difference between the initial absorbance and the absorbance at the subsequent time (10 min). Here, sample refers to the presence of *HMG-CoA* reductase, and control refers to the absence of an enzyme. The extinction coefficient for *NADPH* at 340 nm was 6.22 (mmol/L)^−1^ cm^−1^ (since 2 *NADPH* were consumed in the reaction, its value was 12.44); 0.3 mL is the total volume of the reaction; *V* was the volume of the enzyme used in the assay (mL); 0.6 was the enzyme concentration in mg of protein (mg P)/mL; 0.55 cm was the value of the light path for the plates. The results are presented in µmol/min/mg protein (units/mg P). Identical measurements were performed for the enzyme bound to the carrier. Still, the sample was placed in a dialysis vessel (Pur-A-Lyzer, Sigma-Aldrich), which allowed for the elimination of signals from the high concentration of lipids constituting the lipid carrier.

Inhibition measurements were performed by adding appropriate volumes of fluvastatin to the measuring solutions, up to a final concentration of 10^–5^ mol/L. The results of the inhibition of fluvastatin (sample) are presented in terms of %inhibition compared to that of the control sample in which the *NADPH* oxidation reaction did not occur (control). For this purpose, the following equation was used [54, 55]:

4 $$\%Inhibition= \frac{{\Delta A}_{340/control}- {\Delta A}_{340/sample}}{{\Delta A}_{340/control}} \cdot 100\%$$

UV–Vis measurements were also performed for *HMGR* inserted into the hexosome structure. A sample was taken from the measuring vessel at set time intervals, and the *NADPH* concentration was measured [50].

#### Cyclic voltammetry

Cyclic voltammetry measurements of *NADPH* levels were performed using a potentiostat (CHI Instruments Inc., Austin, TX, USA). In the system, the working electrode was a GC electrode (disk diameter 2 mm), and a silver chloride electrode and a platinum electrode were used as the reference and auxiliary electrodes, respectively (the electrodes were purchased from Redox.me, Norrköping, Sweden). The electrochemical cell was filled with 20 mL of 10 mM PBS buffer as the supporting electrolyte. The system was modified by adding a small vessel with a dialysis membrane (final solution volume of 3 mL), which contained the substrates of interest and a sample of *HMGR* either bound in hexosomes or in the free enzyme solution.

Cyclic voltammograms of the *ABTS*-mediated *NADPH* oxidation were recorded in the potential range of 0.0 to 0.8 V and at a scan rate of 10 mV/s. Kochius et al. reported an efficient NAD(P)^+^ regeneration system using *ABTS* as the mediator, demonstrating the reversibility of mediator electrode processes [56]. Schroeder et al. also reported the regenerative properties of the ABTS radical pair [57]. We use *ABTS* only to improve the development of the oxidation signal of *NADPH*.

The %Conversion of *NADPH* to *NADP*^+^ was determined using the following relationship: [58]

5 $${\%Conversion}_{NADPH/{NADP}^{+}}=\left({X}_{control}-{X}_{t}\right)\cdot 100\%$$

*X*_*control*_ is the absorbance or current density for the control sample (without *HMGR* reductase in the system), and *X*_*t*_ is the value measured at a given time.

## Results and discussion

### Characteristics of HMGR-loaded hexosomes

#### Entrapment of *HMGR* within hexosomes

The entrapment of *HMGR* within hexosomes was assessed at two time points: immediately following the preparation of the nanoparticles and 24 h thereafter. This evaluation aimed to confirm the presence of the protein within the hexosomes in time and its effect on the structure of the lipid nanoparticle. Directly after nanoparticle preparation, the incorporation efficiency was measured to be 31.2%, corresponding to 15.6 μg/mL of protein loaded into the hexosomes. Additionally, after 24 h, the entrapment rate was recorded at 30.2%, confirming the stable incorporation of the *HMGR* within the hexagonal phase carrier.

**Fig. 1:**
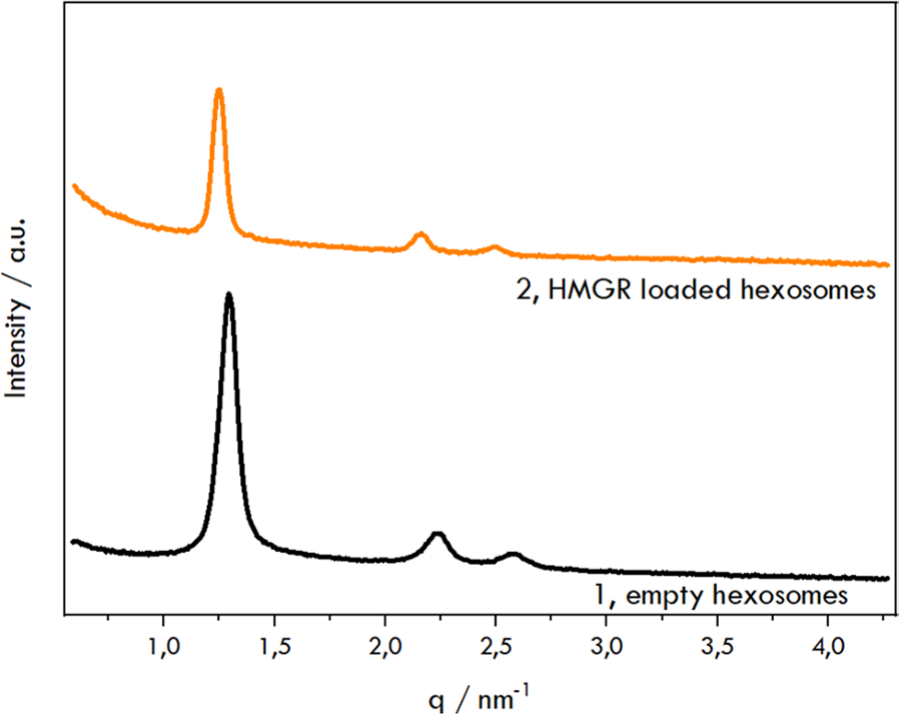
Representative small-angle X-ray scattering diffraction patterns were obtained for hexosomes (1, black line) and proteohexosomes (2, orange line)

Figure 1 presents the diffractograms obtained for the blank formulation and those incorporated with *HMGR*. The non-doped formulation displayed the reflections with relative positions at ratios √1, √3, and √4, which indicate a hexagonal structure with a lattice parameter of 5.5 nm. In comparison, the formulations containing *HMGR*-loaded nanoparticles also displayed reflections consistent with a hexagonal structure; however, a slight increase in the lattice parameter was observed, rising from 5.5 nm to 5.9 nm. Furthermore, the radius of the water channel increased from 2.7 nm to 2.9 nm. Alterations in the unit cell's dimensions also provide indirect evidence of the protein’s accommodation within the hexosomes.

**Fig. 2:**
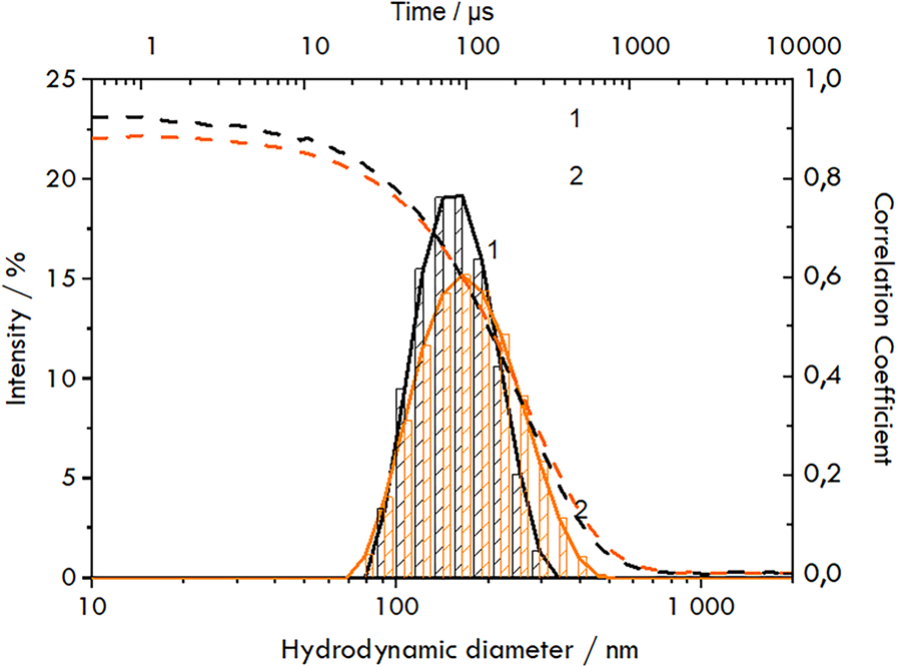
Calculated Gaussian distributions of the hexosomes (1, black) and proteohexosomes (2, orange) sizes determined using the *DLS* method and the correlation coefficient in time for* DLS* measurements (dashed lines—1 and 2)

The presence of protein within the hexosome structure is also reflected in the increase in the particle size measured by *DLS* (Fig. 2) and the increase in the zeta potential (Table 1).

**Table 1:** The hydrodynamic diameters of the obtained structures (hexosomes and proteohexosomes) and their zeta potentials

	Size/nm	PDI	ξ/mV
Hexosomes	150 ± 2	0.067	− 19.2 ± 1.6
Hexosomes with *HMGR*	160 ± 1	0.111	− 6.9 ± 0.5

**Fig. 3:**
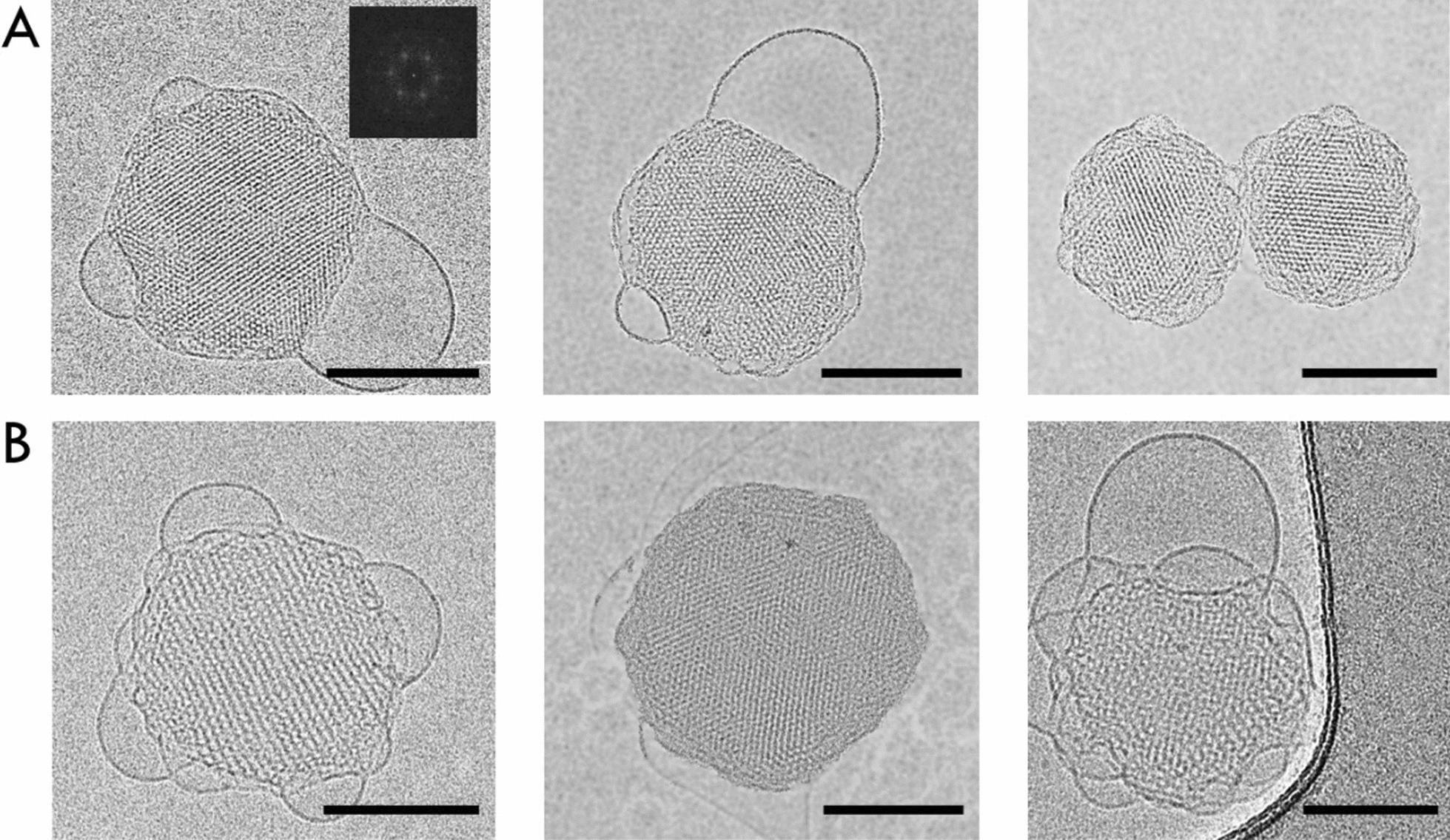
Cryo-TEM images visualizing examples of hexosomes (**A**) and proteohexosomes (**B**). The scale bar is 100 nm. Inset: fast Fourier transform (*FFT*) analysis of the dispersions

The *Cryo-TEM* images (Fig. 3) show the morphology of the obtained nanostructures. The sizes obtained after freezing the samples in liquid nitrogen (T = 90 K) show the same size as in the *DLS* method (hydrodynamic diameter ~ 160 nm). Due to the relatively small size of the enzyme studied (76 kDa), we could not visualize its structure in the hexosomes. Due to the specificity of *HMGR* and the relatively large size of its catalytic site, one can observe wider water channels in the hexosome structures. Large vesicular structures were also observed at the interface of nanoparticles and water. The formation of these structures prevents the lipophilic part of the nanoparticle from contact with water at the water-cubosome interface, as demonstrated by Desmurtas et al. [59].

A close-up representation is presented in the image insets for both structures to better visualize the water channels and their mutual arrangement. Most nanoparticles had near- hexagonal shapes and hexagonal symmetries (confirmed by *FFT*—inset on Fig. 3) [60–62], ordered internal structures, and curved striations similar to the architectures of hexosomes described in the literature. [37–39, 41].

### Monitoring the activity of HMGR in solution and entrapped inside the hexosome by UV–Vis spectrophotometry

**Fig. 4:**
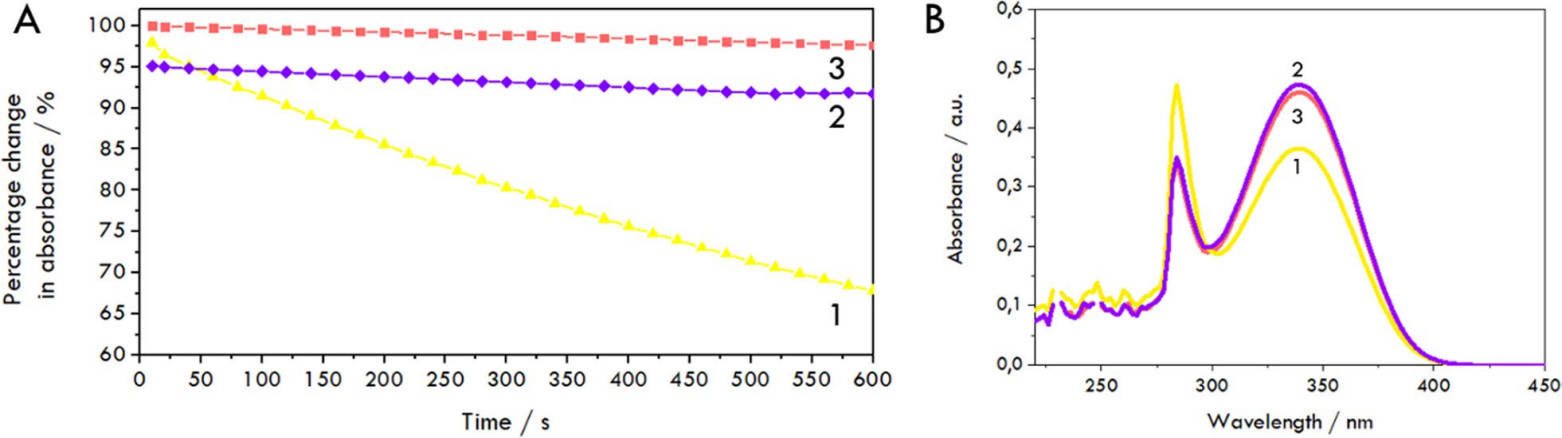
Monitoring *HMGR* (in the form of solution) activity in the solution: **A** The relative change in the absorbance of *NADPH* measured at 340 nm during 10 min of catalytic reaction (1, yellow line). The initial concentration of *NADPH* in the cell was 0.4 mmol/L with 0.2 mmol/L *HMG-CoA*. Absorbance in the presence of fluvastatin (C = 10^–5^ mol/L) in the solution (2, purple line). The absorbance of *NADPH* over 10 min but without *HMGR* in the solution (3, blank sample, red line). **B** The normalized spectra recorded after 10 min of the catalytic reaction (1, yellow line) and absorbance showing inhibition in the presence of 10^–5^ mol/L fluvastatin (2, purple line). The absorbance of *NADPH* during 10 min but without *HMGR* in the solution (3, blank sample, red line)

The changes in the absorbance of *NADPH* measured at 340 nm during 10 min of free *HMGR* catalytic reaction are shown in Fig. 4A. The spectra shown in Fig. 4B were taken at the starting point and after 10 min of the catalytic reaction. The same spectra were recorded in the presence of fluvastatin to show its ability to inhibit the reaction by blocking the catalytic center of *HMGR*. The UV–Vis method has been confirmed to be convenient in determining *NADPH* concentration. In the tests conducted in this work, under the specified conditions, the *LOD* was determined based on the calibration curve in the concentration range of 10^–6^ to 10^–3^ mol/L, and is equal to 1.77·10^–6^ mol/L.

The experiments using the enzyme in the solution confirmed the enzyme’s activity, showing the decrease in the absorbance value at the measured wavelength (yellow curves, Fig. 4). For *NADPH*, two bands are observed—the main one at 340 nm, characteristic of the reduced form, and that at 260 nm, which also occurs for *NADP*^+^. During the reaction, a decrease in the absorbance maximum of the first is observed, while the oxidized form *NADP*^+^ increases in intensity [50]. The inhibition of the catalytic site of the reductase by the statin present in the system confirms the proper functioning of the enzyme (purple curves, Fig. 4). The percentage of inhibition is practically almost 100% (94–97%). Additionally, we extended the measurement to a time of 60 min, demonstrating that the enzyme remains active and the level of *NADPH* used in the sample is high enough for the reaction to keep going for one hour.

**Fig. 5:**
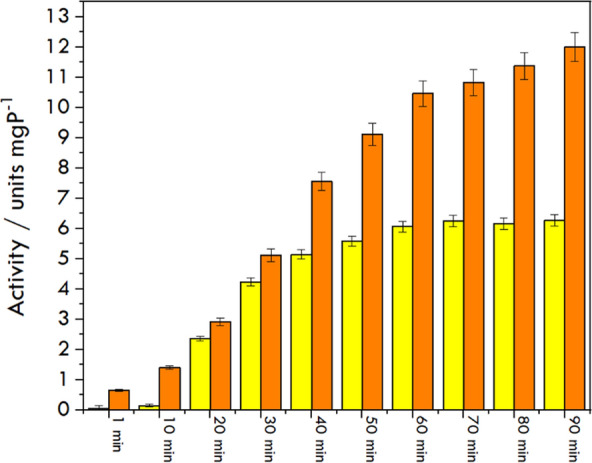
HMGR activity in the solution form (yellow) and incorporated into hexosomes (orange) measured using the UV–Vis method and calculated using Eq. 2

Next, the *HMGR* was incorporated into the hexosomes, and the same reactions were performed with the encapsulated enzyme. UV–Vis measurements were performed by incubating the carriers in the solution containing *HMG-CoA* and *NADPH* at the same concentrations as in the measurements with the enzyme in the solution. To eliminate the interfering effect of the carrier material in the determination of *NADPH*, the hexosomes were separated in a tube with a dialysis membrane, and the decreasing concentration of *NADPH* was measured using the solution outside the tube. The activity of encapsulated *HMGR* was maintained in the 90-min experiment. In the case of the carrier-bound enzyme, a slower initial decrease of *NADPH* levels was observed, related to the time it takes for *NADPH* and *HMG-CoA* to diffuse into the lipid carrier compartment and reach their binding sites. The lag in the reaction progress curves was expected, given the need for substrate/product diffusion into and out of the mesophase, as discussed by Li and Caffrey [36]. We observed the stabilization of the activity of the solution after about one hour of measurement, while for the bound enzyme, it was slower (Fig. 5). The intention of using the carrier is to stabilize the enzyme regardless of the storage temperature and other conditions. This fact has been confirmed in our experiments—binding the enzyme in the carrier allowed for stabilizing it for up to a couple of days. After storing both *HMGR* samples (solution and hexosome-bound) at room temperature (25 °C) without reagents initiating the catalytic reaction, activity tests were performed again within 60 min. The enzyme stored in the hexosomes for 24 h showed higher activity (*A*_*60 min*_ = 2.23 units/mg) than when remaining for the same length of time in the solution environment (*A*_*60 min*_ = 0.91 units/mg). The experiment was repeated after 4 days; the entrapped enzyme in the hexosome showed the same activity value (*A*_*60 min*_ = 2.20 units/mg). However, we observed that the enzyme in the solution sample lost its activity (*A*_*60 min*_ = 0.65 units/mg). The stabilizing role of the lipid carrier was demonstrated. Usually, such proteins have to be stored at very low temperatures (− 70 °C), and very quickly lose their activity at 4 °C (as stated by the manufacturer).

### Monitoring the activity of HMGR in solution and entrapped inside the hexosome by cyclic voltammetry in the presence of *ABTS*

**Fig. 6:**
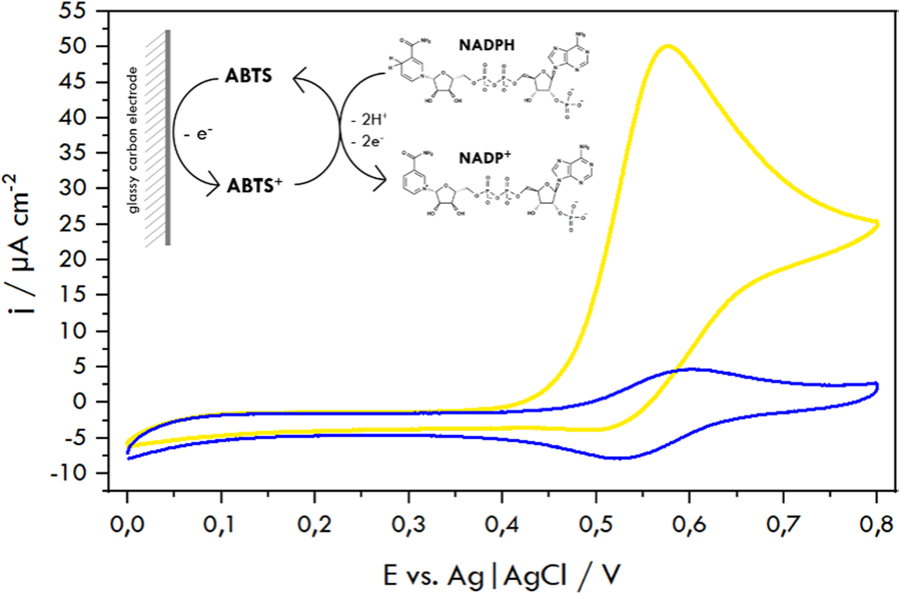
Cyclic voltammograms obtained for 40 μmol/L *ABTS* (1, blue line) and in the presence of 0.4 mmol/L *NADPH* (2, yellow line) in PBS pH 7.4. Scan rate: 10 mV/s. Inset: redox catalysis of *NADPH* and* ABTS* (adapted from) [64].

The *NADPH* levels can be conveniently measured in an alternative way using cyclic voltammetry. *CV* offers lower detection limits (*LOD* = 7.928·10^–8^ mol/L), making it more suitable for detecting redox-active species like *NADPH*, even in complex samples. The addition of *ABTS* in *CV* enhances the detection of *NADPH* by acting as an electron mediator [63], improving the sensitivity and enabling the detection of *NADPH* at lower concentrations, even in the presence of other species like *HMG-CoA* and the enzyme. In our work, we have adapted the methods of determining various substances in the presence of *ABTS* demonstrated by Paice et al. [64]. In the discussed report, we rely on the process of *NADPH* oxidation in the presence of the ABTS mediator, which is presented in Fig. 6 (inset). The *ABTS* voltammogram in the absence of *NADPH* (Fig. 6, blue line) confirms the reversibility of the mediator electrode processes. It is visible that *ABTS* reacts with *NADPH* (in a tenfold excess), which can be observed by the increase of the oxidation current and decrease of the reduction current.

The *HMGR* blank solution was prepared in 10 mM PBS buffer at a concentration of approximately 4·10^–8^ mol/L (the initial sample concentration given by the manufacturer was 0.5–0.7 mg/mL). The *HMG-CoA* reductase solution and the *HMG-CoA* substrate at a concentration of 0.2 mmol/L were placed in a 3 mL dialysis membrane cassette. In the second variant, we added 500 µL of a suspension of hexosomes containing *HMGR* separated from unbound enzyme to the cassette (as described above). Measurements were performed for 0.4 mmol/L *NADPH* and 40 μmol/L *ABTS* in the supporting electrolyte outside the dialysis cassette. Between measurements of the cyclic curves, the solution was stirred at 300 rpm. In the following summary, we compare measurements of the activity and inhibition by fluvastatin of *HMGR* in solution form and incorporated into hexosomes.

**Fig. 7:**
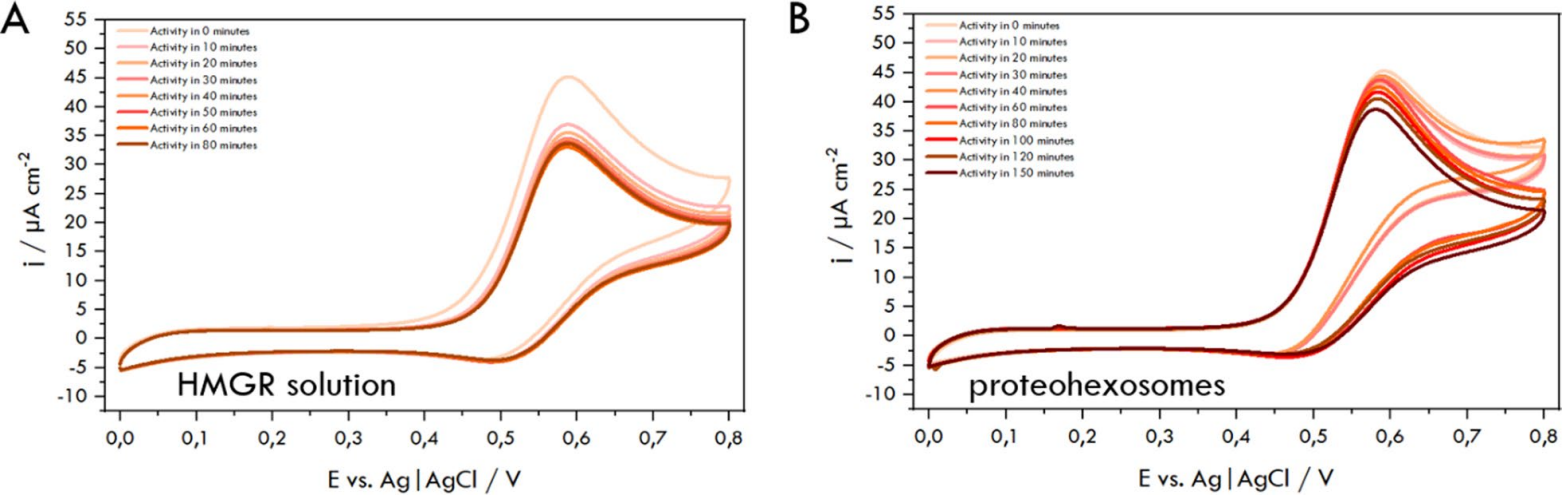
Cyclic voltammograms obtained for *HMGR* solution (**A**) and hexosomes with *HMGR* (**B**) in PBS pH 7.4 in the presence of 0.2 mmol/L *HMG-CoA*, 0.4 mmol/L *NADPH*, and 40 μmol/L *ABTS*. Scan rate: 10 mV/s

Before the initiation of the catalytic reaction (t = 0 min), we observed almost identical *CV* curves, which illustrate *NADPH-ABTS* interactions (*j* = 50.5 μA/cm^2^, Fig. 6) and an oxidation current density of 45.1 μA/cm^2^ (Fig. 7 A and B). The changes in the cyclic voltammograms with the progress of the catalytic reaction of the enzyme both in the free (solution soluble) form and when hosted in the hexosomes, are shown in Fig. 7A and B, respectively. It should be noted that within 10 min, we observed an almost 18% decrease in the *NADPH* oxidation current value for *HMGR* in solution, which agrees with the UV–Vis measurements' data. Comparing the result for *HMGR* entrapped in hexosomes and *HMGR* in the solution, it is evident that the substrates (*HMG-CoA*, *NADPH*) diffuse slower through the hexosome lipid layers when accessing the catalytic site than in the case when *HMGR* remains in the solution.

**Fig. 8:**
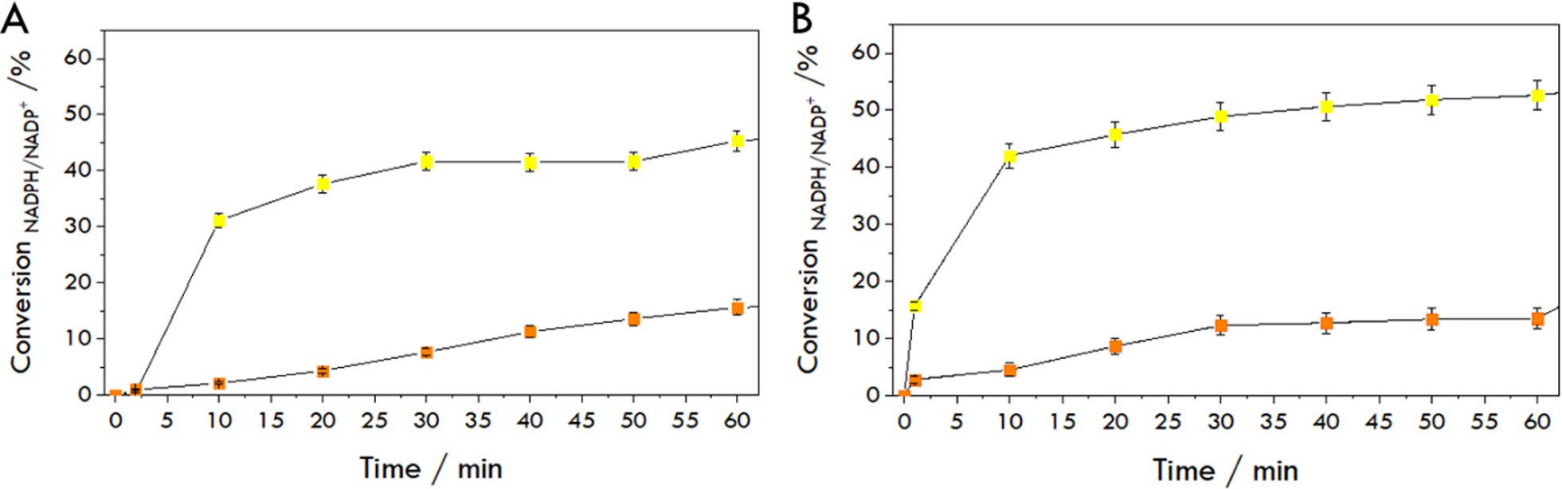
Conversion percentage of *NADPH* to *NADP*^*+*^ in the HMGR-catalyzed reaction monitored spectrophotometrically (**A**) and voltammetrically (**B**) during reduction of *HMG-CoA* to mevalonate in PBS pH 7.4 in the presence of 0.2 mmol/L *HMG-CoA* (1, yellow for *HMGR* solution and 2, orange for *HMGR* entrapped in hexosomes) and 0.4 mmol/L *NADPH* (and 40 μmol/L *ABTS* in the case of *CV*)

The changes in oxidation current recorded during the anodic half-cycle curve were used to determine the time dependencies of the reaction and the activity of the enzyme based on the conversion percentage derived from Eq. 5 (Fig. 8). We observe differences in the time needed for a similar extent of conversion of *NADPH* to *NADP*^+^, which is greater in the case of lipid carriers, similar to the results of UV–Vis measurements. It should be noted that the voltammetric approach is more sensitive to changes in *NADPH* concentration at lower—micromolar levels.

**Fig. 9:**
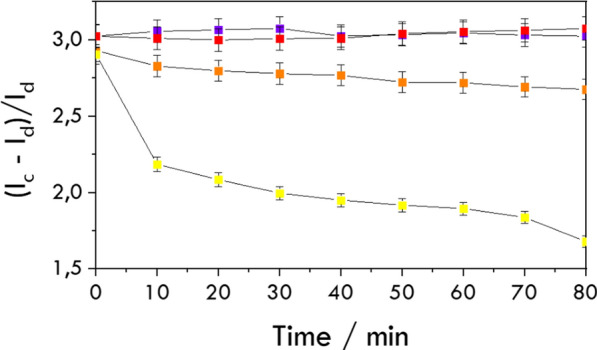
Time dependence of the *ABTS*-related oxidation peak current in the presence (purple and red lines) and absence (yellow and orange lines) of 10^–5^ mol/L fluvastatin recorded for *HMGR* solution (purple and yellow), and hexosomes containing *HMGR* (red and orange) in the presence 0.2 mmol/L *HMG-CoA*, 0.4 mmol/L *NADPH *and 40 μmol/L *ABTS*. I_c_ is the peak current in the presence of *NADPH*, and I_d_ is the diffusion current of* ABTS* in the absence of *NADPH*

In contrast to measuring enzyme activity, the potential for inhibition should be examined by introducing an *HMG-CoA* reductase inhibitor, such as a statin, into the measurement system. The enzyme catalytic current (*I*_*c*_) time dependence was compared to that of the diffusion current (*I*_*d*_). *I*_*c*_ is the current measured during the catalytic process, while *I*_*d*_ is the value of the diffusion current of *ABTS* in the absence of *NADPH* (*I*_*d*_ = 0.365 μA). For the unbound enzyme form (solution *HMGR*), the lack of changes in current indicates that fluvastatin, at a concentration of 10^–5^ mol/L, practically immediately inhibits the catalytic site of *HMG-CoA* reductase (Fig. 9).

In the case of proteohexosomes, the drug takes about 5 to 10 min to reach the catalytic site inside the hexosome, which is the time required to initiate inhibition. This suggests that the enzyme is likely located within the water channels of the structures. The enzyme-catalyzed reaction will have progressed by approximately 30% during this period. The graph shows the inhibition normalized to enzyme activity after 10 min. After this initial period, we observe no further changes in the oxidation current for the reaction. The effects observed in both the solution test and the proteohexosomes are similar after the first 10 min. The inhibition calculated according to Eq. 4 is between 95 and 98% (corresponding to UV–Vis experiments). This suggests that the lipid bilayer of the hexosomes, which acts as the matrix for the reductase, no longer obstructs the drug's access to the enzyme active site.

## Conclusions

This study aimed to evaluate the applicability of lipid nanoparticles, specifically lipid liquid-crystalline nanoparticles known as hexosomes, for encapsulating sensitive proteins, focusing on transmembrane proteins. The goal was to use these nanocontainers to prolong the life and activity of the proteins. The research centered on *HMG-CoA* reductase (*HMGR*), a critical protein involved in cholesterol biosynthesis.

*HMGR* encapsulated in hexosomes was compared to its activity in an aqueous solution. Incorporating *HMGR* into lipid nanoparticles demonstrated improved stability, indicating better protection against denaturation and enhanced longevity as the catalytically active center in the *CoA* transformation process. Hexosomes provide an optimal balance between water and lipids, creating a suitable environment for the enzyme.

Adding *PEG* and Pluronic®F127 polymers made it possible to produce proteohexosomes without relying on sonication. This is beneficial because sonication can be too harsh for many sensitive membrane proteins, whereas smaller drugs generally tolerate such treatment. Additionally, incorporating *PEG* or pegylated lipids as nonionic stabilizers has been shown to stabilize dispersions of non-lamellar liquid crystalline nanoparticles and improve their pharmacokinetic profiles.

The sizes of the new *HMGR* carriers were consistent when measured using *DLS* and *Cryo-TEM*. Small-angle X-ray Scattering (*SAXS*) results indicated that the structures of the nanoparticles, both with and without the protein, were hexagonal. Evidence of protein encapsulation was observed through widened water channels, likely resulting from the presence of the hydrophilic catalytic portion of the protein.

Although the percentage of enzyme entrapment in the nanocontainer was relatively low (with an encapsulation efficiency of about 30%), we have successfully demonstrated a suitable procedure to achieve protein encapsulation. Hexosomes can effectively hold sensitive membrane proteins and shield them from external factors and larger unwanted biomolecules that could interfere with their activity. Significantly, they do not obstruct the penetration of substrates needed for catalytic reactions, as shown by various activity tests. The presence of lipid layers slows the diffusion of reactants to the catalytic site, delaying the time it takes for the substance to reach the catalytic center. Monitoring changes in *NADPH* concentration allowed us to observe variations in *HMGR* activity over time. UV–Vis spectroscopy and *ABTS*-based cyclic voltammetry were suitable for this purpose, with the voltammetric approach exhibiting greater sensitivity to changes in *NADPH* concentration, particularly at low micromolar levels.

A significant finding of this work is that the enzyme housed in hexosomes maintains its full activity for an extended period. Even after several days, when the catalytic reaction begins, the enzyme stored in hexosomes remains active. After 96 h of storage, the addition of *NADPH* initiated the catalytic process, highlighting the advantages of encapsulating the enzyme in a lipidic mesophase environment compared to solubilizing it using the detergent. The detergent—solubilized enzyme stored in aqueous solution at room temperature loses activity much more rapidly, likely due to detergent exposure and denaturation.

We used the *HMGR* as an example of a sensitive protein with complex chemistry, but its activity can be easily monitored using *NADPH* as a marker. This allows us to readily determine the utility of selected inhibitors. The hexosome nanocontainer can assist in the search for other activators and inhibitors of *HMGR*, as well as in the characterization of other oxidoreductases.

When using lipid nanocontainers to encapsulate membrane proteins, it is important to consider their composition and compatibility with cell membranes. This is particularly relevant for applications not only for the protein characterization but also for delivering proteins and peptides needed to address deficiencies or to support the organism in combating microbial or viral attacks. This study demonstrated the potential of lipid liquid-crystalline nanocontainers to store the protein, enhance its stability and activity over time. They can maintain proteins in the active state for a longer duration compared to when they are solubilized in bulk solution. Additionally, these nanocontainers allow for monitoring enzyme activity and evaluating the inhibitory effects of drugs, exemplified here by the effects of fluvastatin.

## Data Availability

The dataset generated during the study is available in the Repository of University of Warsaw (link: 10.58132/MNHH9G).
